# Utility of a thematic network in primary health care: a controlled interventional study in a rural area

**DOI:** 10.1186/1478-4491-3-4

**Published:** 2005-07-22

**Authors:** Maria Jesús Coma del Corral, Pedro Abaigar Luquín, José Cordero Guevara, Angel Olea Movilla, Gerardo Torres Torres, Javier Lozano Garcia

**Affiliations:** 1 Research Unit, Hospital General Yagüe, Burgos, Spain; 2 Department of Nephrology, Hospital General Yagüe, Burgos, Spain; 3 Health Center of Primary Care, Hospital General Yagüe, Burgos, Spain

## Abstract

**Background:**

UniNet is an Internet-based thematic network for a virtual community of users (VCU). It supports a virtual multidisciplinary community for physicians, focused on the improvement of clinical practice. This is a study of the effects of a thematic network such as UniNet on primary care medicine in a rural area, specifically as a platform of communication between specialists at the hospital and doctors in the rural area.

**Methods:**

In order to study the effects of a thematic network such as UniNet on primary care medicine in a rural area, we designed an interventional study that included a control group. The measurements included the number of patient displacements due to disease, number of patient hospital stays and the number of prescriptions of drugs of low therapeutic utility and generic drug prescriptions by doctors. These data were analysed and compared with those of the control center.

**Results:**

Our study showed positive changes in medical practice, reflected in the improvement of the evaluated parameters in the rural health area where the interventional study was carried out, compared with the control area. We discuss the strengths and weaknesses of UniNet as a potential medium to improve the quality of medical care in rural areas.

**Conclusion:**

The rural doctors had an effective, useful, user-friendly and cheap source of medical information that may have contributed to the improvement observed in the medical quality indices.

## Background

The Internet has tremendous potential for the delivery of instructional materials, allowing collaboration among a virtual community of users (VCU). The future success of the Internet in medicine, among other areas, will depend on their ability to enable physicians to participate in continuous medical education at a time and location convenient for them, along with useful communication. Functions such as electronic mail, chat rooms, mailing lists and computer conferencing enable physicians to establish and maintain communication and collaboration with colleagues investigating the same subjects. This encourages creation of virtual networks, such as UniNet, that allow physicians to share experiences and learn from one another [1,2].

The UniNet project (University Network of Thematic Resources for Virtual User Communities: ) is the first real pilot experience that proposes integrated virtual thematic services for a VCU [3]. A VCU is a group of Internet users who share a set of common aspects of the "knowledge society". UniNet began operating by the end of 1996. The UniNet project aims to be universal, free of language or geographical boundaries and open to all the interests represented in the "knowledge society", but it covers mainly scientific, academic and cultural topics [4]. Currently the major emphasis is on medical and health science VCUs.

The UniNet project is based on the voluntary and altruistic cooperative work of scientists and professionals of many countries across five continents. UniNet also intends to supply information and communication channels on the Internet to every member of the "knowledge society". Another important mission of UniNet is to provide the best resources for the inexperienced user, even one who has no previous knowledge of computers [5,6]. UniNet supports a virtual network on Internet, for a community of doctors oriented to supporting the best practices in patient care.

## Goal

Our objective was to evaluate the usefulness of a thematic network such as UniNet, specifically in medical family practice. The goal of the study is to analyse the effects of the thematic network UniNet in the following four outcomes of a primary care medical practice in a rural area: patients' referrals, number of hospital stays, use of prescriptions of drugs of low therapeutic utility and generic drug prescriptions. We tried to quantify the benefits of doctors having access to medical information of high quality, more continuing medical education and easy coordination with specialist physicians.

## Methods

At the end of 1998, we presented a research proposal based on the study – before and after – to connect the doctors from a basic zone of health (BZH), located in a rural area, to UniNet through the public Internet. A grant helped us to provide computer resources and Internet connection to the rural medical center, allowing rural health care professionals to join a VCU at the UniNet network for collaborative work with medical specialists from the General Yagüe Hospital by web, text conference and e-mail.

## Design

We designed a controlled interventional study on family doctors in a rural health area. A before-and-after study was performed with a control group in order to evaluate the educational intervention.

We pursued this study in a BZH in the province of Burgos, with 5700 inhabitants affiliated with our public health system, and eight doctors – seven of them primary care physicians and one paediatric specialist – who care for patients in three different doctors' offices, located in the larger towns of this rural area: a central office and two peripheral doctors' offices. This BZH is located more than 100 km from their reference center, the General Yagüe Hospital, and the road has three mountain passes. A control group was used: an adjacent BZH, with a population of 4378 affiliated to the public health system, five doctors and a similar environment, nutritional habits, etc. The centers of these areas are 7.5 km apart, and they have the same kind of population.

The intervention consisted of the establishment of a local area network (LAN) with free access to the Internet, and access to the UniNet network. Computer equipment was installed at the offices of every rural doctor. These doctors continuously used different services available on the Internet to retrieve information, perform searches in bibliographic databases, etc. The different services used were:

1. The World Wide Web, which was used as a complex entity with information from a variety of source types, including databases accessible on the Web and from the hospital library's collection of Web-based e-journals and databases. Also, at the UniNet site the doctors could find relevant information including courses, a doctoral program, and congresses organized on the network, such as the 3rd Congress of Nephrology on the Internet (CIN2003), organized by us during the the study. In addition, search engines were used to retrieve and store specific information related to the required health field.

2. Electronic mail (e-mail) was used as a tool of private communication by the rural and specialized doctors. We included the use of a mailing list to transmit information to the participants in this study, and tried to encourage their participation in other meetings, such as the online congress.

3. Chat sessions, or text conferencing, was the most relevant tool. Sessions of continuing medical education were planned as updates on diverse medical subjects as requested by the rural doctors. These sessions were provided by means of the simultaneous connection of the specialized doctors involved and the rural doctors in a private channel of text-conferencing.

All these interventions, including the initial training in Internet tools, occurred during a 12-month period. The global coordination of the project was made by the Research Unit of the General Yagüe Hospital. The educational activities were conducted by specialists from diverse medical departments: nephrology, haematology, rheumatology, neurology, gynaecology, etc. The specialists from the Department of Nephrology led the communication with the BZH and took the lead role in coordinating the educational activities.

For these purposes, each doctor had a personal computer installed in his or her office. In addition, these doctors had access to the databases at the hospital's library, as well as other existing ones freely available via the Internet (Pubmed, Embase, etc).

It took nine months to make the site operational. The first three months of the project were spent on administrative efforts, including equipment orders and installation. During the next three months the software was installed and the network and computers were configured. Afterwards, another six months was used for scheduling and training.

The work plan began with the installation of the local area network (LAN) and the computing equipment. Next, an Introduction to Internet course was delivered, and in the following months, the doctors were trained in computer operation and how to use the diverse services available via the Internet: Web, electronic mail, online databases and text conferencing.

Between January and June 2001, when it was agreed that the doctors had had enough training, we launched the first steps of the continuing medical education program, including clinical sessions, bibliographic reviews, conferences to maintain currency, debate, etc., using live communication by text conferencing, hosted on the UniNet network.

Generally we planned and ran a weekly conference session with the hospital's doctors along with those in the BZH location. A program of subjects of interest presented by the rural doctors was developed. The procedure was as follows: a written document related to the item was sent to the participants before the conference via e-mail, so that it could be read by the rural doctors in advance. Later, at a text conference, all the doctors could discuss the subject more effectively. Every session included bibliographic directions and practical questions. In addition, there was continuous interaction between the specialists and the primary care doctors during the week via e-mail, etc.

In order to measure the effectiveness of these procedures with regard to the effect on the patients, the variation of four parameters, corresponding to the years 2000 (before the intervention) and 2001 (the year of the intervention), was compared.

1. Referral to a hospital specialist. This was defined as every consultation of the patients from the Basics Zone of Health (the BZH of the study and BZH control), requested by their family doctor, to the departments of the hospital that corresponded to any of the CIE-10 codes.

2. Number of hospital stays. This means the number of nights that the patient of the BZH stayed in the hospital, occupying a bed.

3. The proportion of prescriptions of drugs of low therapeutic utility (UTB) with regard to the total of prescribed drugs. One considers the crude rate and the standardized rate, adjusted for the active population *versus* retired persons; the Spanish population census and the direct method of standardization were used to adjust the rate. The UTB drugs are found in a list of UTB, described annually by the Directorate of Public Health (INSALUD)

4. Generic drug prescription: the proportion of prescribed generic medicines relative to the total.

All these data were gathered through a blind and independent method with respect to this study by the Directorate of Public Health in Burgos. The doctors of the BZH of the study did not know the evaluation parameters, and the BZH control remained isolated from this investigation.

## Results

The number of consultation visits to specialists for patients of both BZH shows a reduction of the number and rates of derivation in both, but this reduction is more pronounced in the BZH under study, where the intervention took place. The total reduction, measured by difference between the relative risk reduction in the studied BZH and the BZH control was 4.6% in nephrology, 19.36% in all medical specialties and 13.89% in global consultations, as we can see in Table 1.

**Table 1:** Number (N) and rate of consultations per 1000 inhabitants (°/°°) of nephrology specialists to all medical specialties, and total of consultations (medical and surgical specialties) of patients from both centers, relative to the reference hospital. RRR: relative risk reduction.

**Consultations**	**Center**	**Year 2000** **N (°/°°)**	**Year 2001** **N (°/°°)**	**Variation 2001–2000** **N (°/°°)**	**RRR**	**Difference in RRR (Total effect)**
Nephrology	Studied BZH	15 (2.63)	10 (1.75)	-5 (-0.88)	-33.46%	-4.6%
	Control	10 (2.28)	7 (1.6)	-3 (-0.68)	-29.86%	
Medical (including Nephrology)	Studied BZH	701 (122.98)	422 (74.05)	-279 (-48.93)	-39.78%	-19.36%
	Control	475 (108.5)	378 (86.34)	-95 (-22.16)	-20.42%	
Totales (Medical and Surgical Specialities)	Studied BZH	1685 (295.61)	1189 (208.59)	-496 (-87.01)	-29.43%	-13.89%
	Control	1164 (265.87)	983 (224.53)	-181 (-41.34)	-15.54%	

Hospital admissions (Table 2), measured in absolute numbers, rate of derivation by 1000 inhabitants and the average stay all decreased in both BZH with respect to the nephrology department. This rate is lower in the studied BZH than in the BZH control and it does not vary in the studied BZH, while it decreases in the BZH control. The total average hospital stay decreased in the patients from the studied BZH, whereas it increased for the patients in the BZH control.

**Table 2:** Hospitalization of patients in the Department of Nephrology and the total hospitalization of patients for any specialty at the study center and control center: number (N), rate per 1000 inhabitants (°/°°) and average stay (AS). RRR: relative risk reduction in the rate.

**Hospitalization**	**Center**	**Year 2000** **N (°/°°)** **AS**	**Year 2001** **N (°/°°)** **AS**	**Variation 2001–2000** **N (°/°°)** **AS**	**RRR**	**Difference in RRR** **(Total effect)**
Nephrology	Studied BZH	5 (0.87) 8.4 days	4 (0.7) 8.75 days	-1 (-0.17) +0.35 days	-19.54%	0.19%
	Control	10 (2.28) 5.9 days	8 (1.83) 14.25 days	-2 (-0.45) + 8.35 days	-19.73%	

Total	Studied BZH	447 (78.42) 8.44 days	449 (78.77) 7.12 days	-2 (+ 0.35) -1.32 days	0.44%	4.93%
	Control	410 (93.65) 7.38 days	388 (88.62) 8.65 days	-22 (-5.03) + 1.27 days	-5.37%	

With respect to the prescription of drugs of low therapeutic utility (UTB), there was a reduction of 0.47% in the study BZH, whereas in the control BZH the reduction was of 0.04%, as shown in Table 3. The percentage of UTB, adjusted for the active population *versus* retired persons, shows a reduction of 0.87% in the study BZH and an increase of 0.32% in the control BZH. The difference between RRR was a reduction of 15.31%, as shown in Table 4.

**Table 3:** Percentage of prescription of drugs of low therapeutic utility (UTB) in the rural study center and the control center. RRR = relative risk reduction.

**% UTB**	**Year 2000**	**Year 2001**	**Variation 2001–2000**	**RRR**	**Difference in RRR (Total effect)**
Studied BZH	6.75%	6.28%	-0.47%	-6.96%	-6,26%
Control	5.50%	5.46%	-0.04%	-0.72%	

**Table 4:** Percentage of UTB drugs prescribed relative to the active population *versus* retired persons in the study center and the control center. RRR= relative risk reduction.

**Adjusted UTB**	**Year 2000**	**Year 2001**	**Variation 2001 – 2000**	**RRR**	**Difference in RRR (Total effect)**
Studied BZH	8.84%	7.97%	-0.87%	-9.84%	-15.31%
Control	5.85%	6.17%	0.32%	5.47%	

With regard to the proportion of generic drug prescriptions, the results are shown in Table 5. The period of study coincides with an institutional directive to preferentially prescribe generic drugs in order to cut costs. As we can see, both centers increased their generic medicine prescription rate, but the study BZH did so at a greater rate.

**Table 5:** Percentage of generic drug prescriptions compared to the total number of prescribed drugs. RRR = relative risk reduction.

**Generics**	**Year 2000**	**Year 2001**	**Variation 2001–2000**	**RRR**	**Difference in RRR (Total effect)**
Studied BZH	5.3%	8.53%	3.23%	60.94%	36.74%
Control	5.0%	6.21%	1.21%	24.20%	

## Discussion

The results of our study showed positive changes in medical practice, reflected in the improvement of the evaluated parameters in the study BZH compared with the control BZH during the study period. We will now examine the real effect of the intervention on those positive changes; that is to say, the influence that it is possible to attribute to the UniNet network on the improvement of the medical practice indicators. The limitations of our study are related to its design, in that it is quasi-experimental. However, the existence of a control group of similar characteristics permits us to avoid many of these difficulties and lends weight to the results.

The analysis of Dorsch [7] indicates that rural doctors appear to have the same basic information needs as their urban counterparts, and that both groups rely on colleagues and personal libraries as their main sources of information. Rural practitioners, however, tend to make less use of journals and online databases and ask fewer clinical questions, a difference that correlates with geographical and demographic factors. Rural practitioners find many barriers to information access, including lack of time, isolation, inadequate library access, lack of equipment and skills, higher costs and an inadequate Internet infrastructure.

The Internet removes some of the isolation barriers and it has the potential to facilitate communication among rural health professionals and urban specialists. This was the goal of UniNet, and it was specifically the objective of this study. The integration of professionals into a VCU such as UniNet will not totally compensate for the lack of local library services, but electronic communications, such as synchronous text conferencing, provide a way to incorporate learning activities through expert collaboration, with a useful and practical model of continuing medical education. The formation of these virtual learning networks can allow physicians to reflect upon, generalize and discuss the applications of new information with their peers [8]. A common pitfall of many information technology programs in rural health centers is to give priority to building the technical infrastructure rather than focusing on meeting local needs and developing local expertise and ownership [9]. Text conferencing is a long-distance communication tool that is simple, powerful and cheap [10], all reasons why it is a useful and valid tool for the objectives.

Curran et al. [1] consider that the Internet is useful for continuing medical learning because it enables personal one-to-one communications: that is, the doctors, both students and instructors, may communicate with their peers through electronic mail. The Internet enables access to library resources, interactive multimedia tutorials and other related clinical and academic resources. Asynchronous or synchronous group communications allow participation in collaborative discussions with colleagues, instructors and experts via asynchronous computer conferencing or online, real-time chat groups and the like.

The limited Internet usage in our professional environment constituted the main hurdle for the study [11]. When we initiated this study, only one of the doctors used Internet services. It required a relatively long training period to achieve the mandatory prerequisite that most professionals in the BZH develop basic Internet abilities. The motivational level among the clinical staff to use the workstations varied, depending on their age, work style and the presence of a leader who would lead by example, as we found in the rural health center. Regardless of the importance of the information needed, busy clinicians will use the service only if the computer connections and interfaces are convenient and easy to use [12].

The project achieved its goals of ensuring that family doctors had access to good-quality, user-friendly, cost-effective medical information. In addition, the family doctors were assisted by a group of specialists at the hospital who served as mentors. The location of the computer was a key factor in ensuring that it was well used for clinical decision making. The doctors at the BZH under study learned how to search Medline, how to locate and gain access to the best information resources on the Internet and how to request materials through the General Yagüe Hospital library. Recently, the National Library of Medicine has developed a similar project, introducing aids that allow access to bibliographic databases (Medline), through the Internet in rural health centers, with initial success [13].

More than a technical tool to facilitate access, information technology can also serve to build and strengthen communities of like-minded individuals and institutional networks. Therefore it is necessary to form a VCU that allows for information selection. A deluge of information, much of which concerns technologically sophisticated treatments or expensive medications, is not always applicable to a rural health center. Information is relevant and useful if it emphasizes prevention and promotes evidence based, cost-effective treatment strategies [14-16].

The Internet differs from other media in one major way: the communication process is bidirectional, with an information source in the active role and the receiver in a more passive role [17]. Westberg and Miller [18] propose a model in which the academic health center integrates and distributes a wide range of electronic and human resources. This model will require substantial funding for online, full-text journal collections and networked bibliographic databases that may be even more expensive than print collections. The advent of electronic full-text journals may improve the information access of rural health professionals.

According to Marshall [19]: "the timely use of publicly accessible, electronic databases containing bibliographic and full-text information has the potential to assist in the maintenance of health professional competence, decrease the isolation and lack of up-to-date knowledge experienced by health professionals practicing outside of major population centers, and improve the quality of patient care by narrowing the gap between the publication of scientific findings and their application by researchers and clinicians". Searching for medical information via computers is certainly still not the norm [20]. Although computers provide access to an overabundance of up-to-date information, many physicians still find it more convenient to read a textbook or a journal or to ask a colleague. The utility of a VCU such as UniNet lies in its access to biomedical knowledge, colleagues and to faculty [21-24].

## Conclusion

Our study found that providing computer resources and Internet connection to a rural medical center enabled rural doctors to join a VCU hosted by the UniNet network for collaborative work with medical specialists and allowed for access to high-quality medical information. Through this network, rural doctors had an effective, useful, user-friendly and cheap source of medical information, which may be related to the improvements observed in the medical quality indices.

## Competing interests

The author(s) declare that they have no competing interests.

## Authors' contributions

All the authors contributed equally to the design, data collection, data analysis, drafting and completion of this article.
